# Continuity of care and patient-reported experiences in Norwegian general practice: are they linked, and does the measurement of continuity matter?

**DOI:** 10.1093/fampra/cmag025

**Published:** 2026-05-13

**Authors:** Rebecka Maria Norman, Øyvind Bjertnæs, Katrine Damgaard Skyrud, Rortveit Guri, Steinar Hunskaar

**Affiliations:** Division for Health Services, Norwegian Institute of Public Health, Oslo 0213, Norway; Division for Health Services, Norwegian Institute of Public Health, Oslo 0213, Norway; Division for Health Services, Norwegian Institute of Public Health, Oslo 0213, Norway; Norwegian Institute of Public Health, Oslo 0213, Norway; Department of Global Public Health and Primary Care, University of Bergen, Bergen 5009, Norway; National Centre for Emergency Primary Health Care, NORCE Research AS, Bergen 5838, Norway

**Keywords:** continuity of care, general practice, patient experience, primary health care, health care surveys, administrative data

## Abstract

**Background:**

Continuity of care has been associated with improved health and healthcare outcomes. Continuity can be measured using registry-based and patient-reported measures, but how these measures relate to patient-reported experiences is less well understood. The main aim was to assess the association between continuity of care and patient-reported experiences in Norwegian general practice, using registry-based and self-reported measures. A secondary aim was to compare the two continuity measures and examine their agreement.

**Methods:**

A cross-sectional study was conducted combining data from a national survey of patient-reported experiences with individual-level registry data on general practitioner (GP) consultations. Continuity of care was measured using a self-reported indicator of normally seeing one's own GP and a registry-based Usual Provider of Care (UPC) index. Associations with patient-reported experience scale scores were analysed using adjusted linear regression. Agreement between registry-based and self-reported continuity were examined by comparing their distributions and associations within the same study sample.

**Results:**

Both registry-based and self-reported continuity were positively associated with higher patient-reported experience scores across all scales (*P* < 0.001), with stronger associations for self-reported continuity. Associations were stronger among patients with long-term conditions. Agreement between registry-based and self-reported continuity was moderate and asymmetrical: most patients with high registry-based continuity reported seeing their own GP, whereas patients with low registry-based continuity were almost equally likely to report seeing their own GP or other GPs.

**Conclusions:**

The findings underline the importance of continuity of care in general practice. Registry-based and self-reported continuity measures capture related but non-interchangeable aspects of continuity and provide complementary information.

Key messages

- Higher continuity was associated with better patient experiences.
- Associations were stronger for self-reported than registry-based continuity.
- Associations were stronger in patients with long-term conditions.
- Registry-based and self-reported continuity showed moderate agreement.
- The two measures captured related but non-interchangeable aspects.

## Introduction

Continuity of care refers to the ongoing care provided to individuals over time, characterized by coherent and connected experiences that align with the patient's medical needs and personal context [1]. Continuity is considered essential for a high-functioning primary healthcare system [2]. In Norway, general practitioners (GPs) serve as the first point of contact and gatekeepers for specialized care. Within the Regular GP Scheme, patients are listed with a regular GP (RGP), based on the assumption that continuity of care improves health outcomes, partly through fostering strong patient–GP relationships [3]. Relational continuity supports trust, adherence, and patient satisfaction. It is important from a patient's perspective [4–7], especially among older and more vulnerable patients [8] Registry-based studies show generally high continuity in Norway, with geographical variation, whereas several other countries report declining continuity [9, 10].

According to Haggerty, continuity of care comprises informational, management, and relational continuity [1]. Longitudinal continuity refers to seeing the same doctor over time and closely related to relational continuity, as repeated encounters may facilitate development of a therapeutic relationship [2]. Although the relationship is inferred, it is often used as a proxy for relational continuity [11, 12]. Most studies have measured longitudinal continuity using registry-based indices, such as the Usual Provider of Care (UPC) index, which captures the proportion of consultations conducted by the same GP [13], and is a quality indicator in the Norwegian GP scheme [14]. Previous studies using registry data have found associations between high continuity and lower mortality [15], improved medical adherence [16, 17], reduced hospitalization [18–20], and lower emergency service use [20, 21]. However, seeing the same GP does not necessarily imply a strong therapeutic relationship, and registry data do not capture contextual factors or patients’ experiences of continuity [11].

Continuity of care can also be assessed from the patient's perspective using survey data. A recent review identified emerging evidence linking patient-reported continuity to health outcomes, although the findings were weaker and less consistent than those from registry data. The authors highlighted the need for larger studies combining administrative and patient-reported continuity measures [22].

While continuity has been linked to clinical outcomes such as mortality and hospitalization, fewer studies have examined its association with patient-reported experience measures (PREMs), which assess patients’ experiences of care, rather than health outcomes, including access, communication, and trust [23]. PREMs provide a direct assessment of patients’ experiences of care, including relational aspects that may be influenced by continuity of care. Previous studies have compared administrative and patient-reported measures of continuity of care. Bentler et al. [11] compared claims-based continuity indices with patient-reported continuity and found limited agreement between the two approaches. A UK-based study [12] examined practice-level continuity, measured using the UPC index, and compared it with patient-reported continuity from the GP Patient Survey, reporting an association between the measures. However, these studies used different populations and levels of analysis, and few studies have compared registry-based and self-reported continuity measures in the same population or examined how each relates to patient-reported experiences. This is especially relevant in high-continuity settings such as Norway, where patients are registered with an RGP and continuity levels are generally high [9]. The main aim of this study was to assess the association between continuity of care and patient-reported experiences, using both registry-based and self-reported measures. A secondary aim was to compare the two continuity measures and examine their agreement.

## Methods

### Setting

In the Norwegian RGP scheme, over 99% of residents are registered with a specific RGP [24]. Most GPs are self-employed and work in group practices, typically organized in small units of 2–5 GPs [25]. If the RGP is unavailable, patients often consult other GPs within the same practice. Patients cannot be refused by GPs and may change RGP up to twice a year. Care is generally provided within the same practice and electronic system.

### Sampling and data collection

This study is a secondary analysis of a national survey of patient-reported experiences with general practice conducted between 5 December 2023 and 3 April 2024, with linkage to registry data for the same study population [26]. The survey was administered by the Norwegian Institute of Public Health (NIPH). The inclusion criteria were patients aged 18 and older who had at least one consultation with a GP in the last 12 months. In each of Norway's 356 municipalities (2023), up to 400 eligible patients were randomly selected; where fewer were eligible, all were included. Eligible patients received a digital invitation with electronic response options and reminders. The invited sample was drawn from the national RGP scheme population. To minimize misclassification due to permanent change of RGP, the eligible study sample consisted of invitees who had ≥3 face-to-face GP consultations in the 24 months preceding data collection and had been listed with the same RGP for ≥12 months before the survey. Survey respondents represent those in the eligible study sample who completed the questionnaire.

### Survey data

#### Patient-reported experience measures

We used three subscales from the Norwegian patient experiences with the GP questionnaire (PEQ-GP): *Assessment of GP* (8 items), *Accessibility* (2 items), and *Enablement* (3 items). *Assessment of GP* and *Enablement* capture relational aspects of care, while *Accessibility* reflects acceptability of waiting times. Items were scored on a five-point response scale [27]. Subscale scores were calculated by transforming the five-point response scale to a 0–100 scale and then computing the mean of available item scores, provided that at least 50% of the items within the subscale were completed.

#### Self-reported continuity of care

Self-reported continuity was measured with the survey item: “Do you normally see your own GP when you have an appointment?” Response options were: “Yes”, “No—a permanent substitute”, “No—different doctors”, and “Not applicable”. The two “No” categories were combined into “normally see other GPs.” Self-reported continuity was then dichotomized as “normally see own GP” vs “normally see other GPs.” These groups are referred to as “own GP” and “other GPs”.

### Registry data

#### Registry-based continuity of care

Registry-based continuity of care was measured using the UPC index, which measures the proportion of all GP consultations conducted with the patient's RGP. Registry data were obtained from the Norwegian Control and Payment of Health Reimbursements Database (KUHR) for all patients in the eligible study sample. The UPC index was calculated as the proportion of face-to-face consultations with the patient's RGP during the previous 24 months preceding the survey [12, 18]. UPC values are on a scale of 0 to 1 (1 = all consultations by the patient's RGP). The UPC index was categorized into three groups: low (≤0.40), medium (>0.40–0.70), and high (>0.70–1.00).

#### Patient and municipality characteristics

Patient characteristics were obtained from registry data and included sex, age, years on the GP's list, number of diagnoses, and number of consultations in the past 24 months (from KUHR and The Norwegian General Practitioner Register). Socioeconomic information from Statistics Norway comprised education, household income (categorized into three groups based on average income after taxes per consumption unit, EU-60 scale), and country of birth (categorized into regions, as shown in tables). Municipal characteristics were measured using Statistics Norway's centrality index, ranging from 1 (most central) to 6 (least central), and the municipalities were grouped into three categories accordingly [28]. Municipality centrality was included for descriptive purposes only. Self-reported data from the survey included the number of long-term conditions, physical and mental health, and time since the last contact with a GP.

### Analysis

We conducted descriptive analyses (counts, percentages, and means where appropriate) for the invited sample, the eligible study sample, and the survey respondents. Associations between continuity of care and patient-reported experiences were examined using linear regression analyses, with patient-reported experience subscales as dependent variables (0–100; higher scores indicate better experiences). Self-reported continuity and registry-based continuity were analysed in separate models. Two regression models were specified: a minimally adjusted model (age, sex, municipality) and a fully adjusted model additionally including education, household income, region of birth, self-reported physical and mental health, and number of long-term conditions. For self-reported continuity, the reference group was respondents reporting “own GP,” and for registry-based continuity, the reference category was high continuity (UPC > 0.7). Multicollinearity was assessed using variance inflation factors. The fully adjusted models were repeated stratified by long-term condition status, and continuity × long-term condition interactions were tested using Wald tests.

Agreement between registry-based and self-reported continuity was examined using Spearman's correlation, cross-tabulation, and logistic regression. We also examined characteristics of respondents in the low UPC group (≤0.4), stratified by self-reported continuity. The logistic regression model was adjusted for age, sex, education, household income, region of birth, self-reported physical and mental health, and municipality.

All analyses were conducted using R version 4.4.1 (R Foundation for Statistical Computing, Vienna, Austria).

## Results

The invited sample comprised 137 629 patients. After excluding patients with fewer than three face-to-face GP consultations in the 24 months preceding data collection, and those who had been listed with the same RGP for less than one year prior to the survey, the eligible study sample comprised 97 318 patients. Of these, 43 486 (44.7%) responded to the survey. Respondents were predominantly women and were born in Norway. UPC scores were approximately comparable between respondents and the eligible study sample (Table 1).

**Table 1:** Characteristics of the invited sample, eligible study sample, and survey respondents.

	Invited study sample (*N* = 137 629)	Eligible study sample (*n* = 97 318)	Survey respondents (*n* = 43 486)
	*n* (%)	*n* (%)	*n* (%)
**Sex**			
Female	75 895 (55.1)	55 456 (57.0)	25 931 (59.6)
Male	61 734 (44.9)	41 862 (43.0)	17 555 (40.4)
**Age in years**			
18–54	67 064 (48.7)	43 771 (45.0)	17 264 (39.7)
55–69	36 486 (26.5)	26 724 (27.5)	15 275 (35.1)
70–79	22 292 (16.2)	17 375 (17.9)	8600 (19.8)
80+	11 787 (8.6)	9448 (9.7)	2347 (5.4)
**Household income**			
Low (decile 1–2)	40 731 (29.6)	29 040 (29.8)	9497 (21.8)
Medium (decile 3–7)	68 865 (50.0)	49 316 (50.7)	23 833 (54.8)
High (decile 8+)	27 645 (20.1)	18 955 (19.5)	10 152 (23.3)
**Education**			
Short (none, primary school)	34 322 (24.9)	24 741 (25.4)	8051 (18.5)
Medium (high school, vocational school)	62 883 (45.7)	45 546 (46.8)	20 945 (48.2)
Long (college/university)	36 980 (26.9)	25 345 (26.0)	14 032 (32.3)
Missing	3444 (2.5)	1686 (1.7)	458 (1.1)
**Country of birth**			
Norway	120 545 (87.6)	86 114 (88.5)	39 412 (90.6)
Nordic (except Norway)	2099 (1.5)	1442 (1.5)	752 (1.7)
EU (except Nordic)	5717 (4.2)	3776 (3.9)	1487 (3.4)
Europe (except EU and Russia)	1834 (1.3)	1023 (1.1)	351 (0.8)
Africa	1698 (1.2)	1150 (1.2)	291 (0.7)
Asia with Turkey	4444 (3.2)	3141 (3.2)	898 (2.1)
USA, Canada and Oceania	411 (0.3)	289 (0.3)	146 (0.3)
America (except USA and Canada)	560 (0.4)	381 (0.4)	148 (0.3)
Missing	321 (0.2)	2 (0.0)	1 (0.0)
**No. of personal GP consultations last 24 months**			
<3	23 378 (17.0)	–	–
3–5	40 875 (29.7)	35 581 (36.6)	15 154 (34.8)
6–10	42 314 (30.7)	36 810 (37.8)	16 935 (38.9)
11+	28 892 (21.0)	24 927 (25.6)	11 397 (26.2)
Missing	2170 (1.6)	–	–
**Duration of years with the same GP**			
0–1	18 846 (13.7)	–	–
1–2	19 305 (14.0)	15 972 (16.4)	6755 (15.5)
3–4	24 297 (17.7)	20 087 (20.6)	8827 (20.3)
5–10	36 046 (26.2)	29 498 (30.3)	13 143 (30.2)
11+	39 135 (28.4)	31 761 (32.6)	14 761 (33.9)
**Municipality centrality**			
Least central (centrality: 5–6)	73 761 (53.6)	51 277 (52.7)	21 929 (50.4)
Moderately central (centrality: 3–4)	48 390 (35.2)	35 105 (36.1)	16 361 (37.6)
Most central (centrality: 1–2)	15 478 (11.2)	10 936 (11.2)	5196 (11.9)
**Time since last contact with GP^a^**			
0–3 months ago			34 997 (80.5)
4–6 months ago			5485 (12.6)
More than 7 months ago			2908 (6.7)
Missing			96 (0.2)
**No. of self-reported long-term conditions^a^**			
0			9613 (22.1)
1			14 723 (33.9)
2			11 015 (25.3)
3+			7894 (18.2)
Missing			241 (0.6)
**Self-reported physical health^a^**			
Very poor/rather poor			3609 (8.3)
Neither good nor poor			13 102 (30.1)
Rather good/very good			26 401 (60.7)
Missing			374 (0.9)
**Self-reported mental health^a^**			
Very poor/rather poor			2005 (4.6)
Neither good nor poor			8713 (20.0)
Rather good/very good			32 360 (74.4)
Missing			408 (0.9)
**Self-reported continuity^a^**			
Normally see other GPs			5578 (12.8)
Normally see own GP			37 695 (86.7)
Missing			213 (0.5)
**Registry-based continuity, UPC score^b^**	Mean: 0.74 (SD = 0.27)	Mean: 0.74 (SD = 0.25)	Mean: 0.76 (SD = 0.24)
	Median: 0.80 (Q1–Q3: 0.55–1.0)	Median: 0.80 (Q1–Q3: 0.58–1.0)	Median: 0.80 (Q1–Q3: 0.6–1.0)
**UPC-categories**			
Low (≤0.4)	19 162 (13.9)	13 333 (13.7)	5238 (12.0)
Medium (0.4–0.7)	33 446 (24.3)	24 517 (25.2)	10 447 (24.0)
High (>0.7)	82 851 (60.2)	59 468 (61.1)	27 801 (63.9)

^a^Self-reported variables from the survey.

^b^UPC values are on a scale of 0 to 1 (1 = all consultations by the patient's regular GP).

Statistical differences were observed between survey respondents and the eligible study sample for all variables (*χ*^2^ tests for categorical variables; *t*-test for the UPC score; all *P* < 0.001).

### Association between continuity and patient-reported experiences

Mean patient-reported experience scale scores were higher with higher continuity of care across both continuity measures, with the largest differences for *Enablement* (Table 2; all *P* < 0.001).

**Table 2:** Mean patient-reported experience scores by registry-based and self-reported continuity.

Scales/items^a^	Registry-based continuity—UPC^a^			Self-reported continuity^b^	
	Low	Medium	High	Other GPs	Own GP
	Mean (SD)	Mean (SD)	Mean (SD)	Mean (SD)	Mean (SD)
**Assessment of GP**	**74.9 (19.2)**	**78.7 (18.2)**	**81.7 (16.6)**	**68.7 (19.6)**	**81.9 (16.4)**
GP takes you seriously	78.8 (22.5)	82.8 (21.2)	85.8 (19.4)	72.7 (23.7)	86.0 (19.2)
GP spends enough time with you	70.3 (25.1)	74.9 (23.6)	78.4 (22.2)	62.6 (25.7)	78.7 (21.9)
GP talks to you in a way you understand	81.1 (19.8)	84.6 (18.9)	87.4 (17.1)	76.2 (21.0)	87.4 (17.0)
GP is professionally competent	79.4 (20.0)	82.4 (19.0)	84.8 (17.4)	74.1 (20.8)	85.0 (17.3)
GP shows interest in your situation	76.0 (23.4)	79.7 (21.9)	82.9 (20.2)	69.2 (24.3)	83.1 (20.0)
GP includes you as much as you would like in decisions concerning you	75.2 (22.5)	79.0 (21.0)	82.2 (19.6)	69.2 (23.3)	82.3 (19.4)
GP provides sufficient information about health problems and treatment	72.5 (22.9)	76.0 (21.8)	79.3 (20.3)	65.9 (23.8)	79.5 (20.1)
GP provides sufficient information about use/side effects of medication	62.2 (28.0)	66.5 (26.7)	70.2 (25.1)	55.1 (28.0)	70.3 (25.1)
**Accessibility**	**56.5 (28.1)**	**60.0 (27.6)**	**66.3 (26.2)**	**48.9 (28.4)**	**65.8 (26.2)**
Waiting time for your last urgent appointment acceptable	65.2 (31.7)	67.8 (31.2)	73.2 (29.2)	58.3 (33.3)	72.9 (29.1)
Waiting time for appointments that are not urgent acceptable	48.6 (30.8)	52.9 (30.1)	60.5 (28.9)	40.1 (30.2)	59.8 (28.9)
**Enablement**	**65.6 (23.3)**	**69.3 (22.1)**	**73.1 (20.5)**	**58.7 (23.5)**	**73.2 (20.4)**
Contact with GP make you better able to understand your health problems	67.8 (24.5)	71.7 (23.3)	75.2 (21.7)	60.9 (25.1)	75.4 (21.6)
Contact with GP make you better able to cope with your health problems	65.2 (25.3)	68.9 (24.1)	72.9 (22.6)	58.2 (25.5)	72.9 (22.5)
Contact with GP better helps you to stay healthy	63.6 (25.4)	67.3 (24.5)	71.3 (22.9)	57.0 (25.5)	71.3 (22.9)

^a^UPC values are on a scale of 0 to 1 (1 = all consultations by the patient's regular GP), categorized as low: ≤0.4; medium: 0.4–0.7; high: >0.7.

^b^Self-reported continuity categories were “other GPs” vs. “own GP”.

Scores are presented as mean (SD) on a 0–100 scale, with higher scores indicating better experiences.

Mean differences across continuity groups were statistically significant (one-way ANOVA for UPC categories; independent-samples *t*-tests for self-reported continuity; all *P* < 0.001).

Table 3 shows the fully adjusted regression estimates for the associations between continuity and patient-reported experience scales. Lower continuity was consistently associated with lower scores across all scales, with stronger associations for self-reported than for registry-based continuity. Regression coefficients differed across scales and continuity categories. Results were similar in minimally and fully adjusted models, indicating robust associations. In subgroup analyses stratified by long-term condition (1+ vs. none), the associations were stronger among patients with long-term conditions than among those without (*p* for interaction < 0.001; Supplementary Table S1).

**Table 3:** Associations between continuity and patient-reported experience scales (0–100, higher = better), adjusted for sociodemographic variables. Negative coefficients represent lower patient-reported experience scale scores compared to the reference group.

Continuity	Assessment of GP coefficient [95% CI]	Accessibility coefficient [95% CI]	Enablement coefficient [95% CI]
Registry-based: Medium UPC (ref. High)^a^	−2.77 [−3.17, −2.38]	−4.14 [−4.75, −3.53]	−3.49 [−4.0, −2.99]
Registry-based: Low UPC (ref. High)^a^	−6.27 [−6.80, −5.73]	−5.92 [−6.74, −5.10]	−7.02 [−7.71, −6.33]
Self-reported: Other GPs (ref. own GP)	−12.72 [−13.21, −12.22]	−13.73 [−14.51, −12.96]	−13.81 [−14.45, −13.16]

^a^UPC values range from 0 to 1 and were categorized as low (≤0.40), medium (0.40–0.70), and high (>0.70).

Registry-based and self-reported continuity were analysed in separate models.

Models were adjusted for age, sex, education, household income, region of birth, municipality, self-reported physical and mental health, and number of long-term conditions.

Number of observations (*N*) and explained variance (*R*^2^): Registry-based continuity (UPC): *N* = 39 538–42 970 (Adj.*R*^2^ = 0.10–0.13); Self-reported continuity: *N* = 39 059–42 454 (Adj.*R*^2^ = 0.13–0.15).

All coefficients were statistically significant (two-sided *P* < 0.001); 95% confidence intervals are shown.

Interaction by long-term condition status was significant for all outcomes for both registry-based and self-reported continuity (*p*-interaction < 0.001). Stratified estimates are provided in Supplementary Table S1.

### Association between registry-based and self-reported continuity

We found a moderate positive correlation between the registry-based UPC index and self-reported continuity (“own GP”) (Spearman's *r* = 0.42, *P* < 0.001). As shown in Table 4, the association was asymmetric: reporting “own GP” was strongly concentrated in the high UPC category, whereas in the low UPC category respondents were almost equally likely to report “own GP” or “other GPs”.

**Table 4:** Association between registry-based (UPC categories) and self-reported continuity.

	Self-reported continuity	
Registry-based continuity	Other GPs	Own GP
UPC-categories^a^	*n* (%)	*n* (%)
Low	2673 (51.5)	2513 (48.5)
Medium	2095 (20.2)	8297 (79.8)
High	810 (2.9)	26 885 (97.1)

^a^UPC values are on a scale of 0 to 1; UPC values are categorized as low: ≤0.4; medium: 0.4–0.7; high: >0.7.

Percentages are row percentages.

*χ* ^2^ test of independence, *P* < 0.001.

In adjusted logistic regression analyses, higher UPC was significantly associated with reporting “own GP” (OR per 0.1 increase = 1.70, 95% CI: 1.68–1.73, *P* < 0.001).

Table 5 shows the characteristics of respondents in the low UPC group (≤0.40), restricted to those with valid self-reported continuity (*n* = 5186). Among patients with low UPC who reported “own GP”, had fewer GP consultations, longer duration with the same GP, more recent GP contact, better physical and mental health, and GP in a more central municipality. Mean UPC was slightly higher among those reporting “own GP” (0.30 vs. 0.26; *P* < 0.001).

**Table 5:** Characteristics of respondents in the Low UPC group, by self-reported continuity.

	Other GPs^a^ (*n* = 2673)		Own GP^a^ (*n* = 2513)		*P*-value
	*n*	%	*n*	%	
**Sex**					0.704
Female	1621	60.6	1510	60.1	
Male	1052	39.4	1003	39.9	
**Age (years)**					0.520
18–54	1243	46.5	1155	46.0	
55–69	856	32.0	822	32.7	
70–79	436	16.3	426	17.0	
80+	138	5.2	110	4.4	
**Education**					0.504
Short (none, primary school)	520	19.5	496	19.7	
Medium (high school, vocational school)	1292	48.3	1180	47.0	
Long (college/university)	816	30.5	803	32.0	
Missing	45	1.7	34	1.4	
**Household income**					0.787
Low	582	21.8	563	22.4	
Medium	1453	54.4	1366	54.4	
High	638	23.9	583	23.2	
**Region of birth**					0.588
Norway	2408	90.1	2258	89.9	
Europe, North America, Oceania	185	6.9	188	7.5	
Africa, Asia, South America	80	3.0	67	2.7	
**No. of GP consultations last 24 months**					<0.001
3–5	901	33.7	1085	43.2	
6–10	1036	38.8	885	35.2	
11+	736	27.5	543	21.6	
**Duration of years with the same GP**					<0.001
1–2	684	25.6	491	19.5	
3–4	746	27.9	578	23.0	
5–10	761	28.5	832	33.1	
11+	482	18.0	612	24.4	
**Time since last contact with GP^b^**					<0.001
0–3 months ago	1915	71.8	1856	74.1	
4–6 months ago	393	14.7	416	16.6	
More than 7 months ago	360	13.5	234	9.3	
**No. of self-reported long-term conditions^b^**					<0.035
0	654	24.6	695	27.8	
1	934	35.2	874	34.9	
2	625	23.5	564	22.5	
3+	444	16.7	369	14.7	
**Self-reported physical health^b^**					<0.001
Very poor/rather poor	242	9.1	146	5.9	
Neither good nor poor	844	31.8	710	28.5	
Rather good/very good	1567	59.1	1638	65.7	
**Self-reported mental health^b^**					<0.011
Very poor/rather poor	138	5.2	109	4.4	
Neither good nor poor	583	22.0	480	19.2	
Rather good/very good	1929	72.8	1908	76.4	
**Municipality centrality**					<0.001
Least central (centrality: 5–6)	2052	76.8	1599	63.6	
Moderately central (centrality: 3–4)	466	17.4	714	28.4	
Most central (centrality: 1–2)	155	5.8	200	8.0	

^a^Self-reported continuity was categorized as “other GPs” vs. “own GP”.

^b^Self-reported variables from the survey.

*P*-values from *χ*^2^ tests.

## Discussion

This study found a consistent positive association between continuity of care and patient-reported experiences in Norwegian general practice. Continuity of care, as measured by the UPC index, was high in this study in an international context, with only 12% of survey respondents having low continuity (UPC ≤0.4), consistent with previous Norwegian studies and contrasting with declining continuity reported elsewhere [10]. Previous registry-based studies have shown that higher continuity of care is associated with better patient and health service outcomes [15–21]. Consistent with this, we found that both registry-based and self-reported continuity were associated with more favourable patient-reported experiences in general practice. In particular, continuity was associated with higher patient-reported *Enablement*, which includes items about the GP's influence on patients’ understanding of their condition, coping ability, and capacity to stay healthy, all relevant for long-term health outcomes. Patient-reported experiences have previously been linked to improved adherence, clinical outcomes, and patient safety [29, 30]. Together, this pattern suggests a pathway through which continuity of care may influence health and healthcare service outcomes. This is in line with earlier studies, describing how repeated contact with the same GP fosters trust, empathy, and knowledge between the patient and GP, thereby enhancing care quality and health outcomes [31]. The associations were somewhat stronger among patients with long-term conditions, highlighting the importance of sustained continuity for this group. Previous research indicates that patients with chronic conditions benefit most from continuity of care [15]. In an earlier national survey, patients with long-term conditions tended to report slightly poorer experiences than those without such conditions [32]. This pattern may also reflect differences in care organization: patients with long-term conditions have more frequent, planned contacts with their RGP, whereas patients without long-term conditions may be more dependent on *ad hoc* access and therefore more often see other GPs.

We found that 97.1% of patients with high UPC reported “own GP”. However, almost one-third of those reporting “own GP” had low or medium UPC. The asymmetric association between the two measures indicates that they capture related, but non-interchangeable, aspects of longitudinal continuity. Notably, patients with low registry-based UPC who nevertheless reported “own GP” had a longer relationship with the same GP and fewer health-related needs (fewer consultations in the last 24 months, better health). They were more likely to live in central municipalities. These characteristics may partially compensate for fragmented consultation patterns in recent years and suggest that patients may experience relational continuity even when registry-based measures indicate low continuity.

Continuity of care is experienced by patients rather than being directly observable. According to Haggerty et al. [1], continuity is the degree to which a series of discrete healthcare events is experienced as coherent, connected, and consistent with the patient's medical needs and personal context. Within this framework, relational continuity refers to the ongoing therapeutic relationship between patient and provider. Registry-based measures, such as the UPC index, do not capture the context in which consultations with the GPs occur, for example, whether patients are offered choices, prioritize shorter waiting times, or are allocated a substitute GP due to availability or absence. Previous studies indicate that patients may accept or choose to consult another GP to improve access, particularly for less complex or time-sensitive problems, without necessarily experiencing a loss of continuity [33, 34]. Seeing several different substitute GPs may be experienced differently from repeatedly seeing the same substitute, and such contextual differences are not reflected in registry data but may influence how continuity of care is experienced [35]. In addition, patients who frequently consult the same substitute GP over time due to the limited availability of their RGP may come to perceive this GP as their RGP. The mechanisms, predictors, and consequences of the gap between formally assigned and subjectively perceived continuity warrant further investigation. This may explain why self-reported continuity, which reflects patients’ perceived continuity with their RGP, showed stronger associations with patient-reported experiences than the registry-based UPC index in our study. This underscores the need for caution when interpreting registry-based measures, such as the UPC index, as proxies for relational continuity. Future studies may explore the use of more comprehensive patient-reported continuity instruments, such as the Nijmegen Continuity Questionnaire [36]. However, single-item measures may be advantageous in large and thematically broad population-based surveys, where brevity is important, and single-items may still capture meaningful differences in self-reported continuity.

Self-reported continuity was defined as reporting consultations with one's own RGP, while responses indicating consultations with other GPs, including permanent substitutes, were classified as not having continuity. Patients reporting consultations with a permanent substitute may still experience some degree of continuity, although not with their registered GP. In our data, patients reporting consultations with their own GP generally had higher experience scores than those reporting consultations with other GPs, including permanent substitutes. The group reporting consultations with several different doctors was small (approximately 2% of respondents). Our primary definition focused on continuity with the RGP to align the self-reported measure with the registry-based UPC measure, which reflects consultations with the RGP. Alternative definitions, such as continuity with the most frequently seen GP, may be explored in future studies.

Patient-reported experiences primarily capture relational aspects of general practice, and a strong patient–GP relationship is a key goal of continuity of care. Repeated contact with the same GP can strengthen trust, communication, and mutual understanding [1, 37], which are reflected in the *Assessment of GP* and *Enablement* subscales. The stronger associations observed for the self-reported “own GP” measure likely reflect closer alignment in perspective and time frame, as both self-reported continuity and patient-reported experiences are based on patients’ recent experiences of care. In contrast, the UPC index summarize continuity over the previous 24 months and may not reflect patients’ perceptions of care at the time of the survey. Self-reported continuity reflects the current or typical care situation but may be influenced by recall bias or generalized satisfaction and does not necessarily represent a superior measure of continuity. Hence, registry-based and self-reported measures capture complementary aspects of continuity, providing different, non-interchangeable information.

### Implications

Continuity of care was positively associated with better patient-reported experiences, highlighting its importance in a well-functioning general practice system. In countries with a list-based system, such as the Norwegian RGP Scheme, this underscores the need to monitor variation in continuity and to address groups with persistently lower continuity. Given that continuity of care was strongly associated with *Enablement* and *Assessment of GP*, strengthening patients’ ability to consult their own RGP regularly should remain a priority. New models of care that affect access to and continuity with the RGP should be carefully designed and evaluated. Such models may weaken face-to-face continuity, but may also, depending on organization and use, allow the RGP to prioritize problems where continuity is most important. Registry-based and self-reported continuity capture related but not identical aspects of longitudinal continuity. Quality indicators of continuity of care based on registry data, such as the UPC index, do not fully capture patient-reported continuity of care. Patient-reported measures may therefore complement registry-based measures. Moreover, the stronger associations among patients with long-term conditions highlight the importance of continuity for this group. Continuity is an important consideration in reforms addressing patients with chronic conditions in general practice. Finally, further research is needed to explore how different forms of continuity interact over time and relate to patient-reported outcome measures (PROMs) and provider-reported continuity of care.

### Strengths and limitations

A major strength of this study is the use of both registry-based and self-reported measures of continuity of care, which provide complementary longitudinal perspectives. Few previous studies have linked these data sources. The large, nationally representative sample further strengthens the generalizability of the findings. However, this study has some limitations. Common method bias cannot be ruled out, as both patient-reported continuity and patient-reported experiences were based on self-reported data.

Reverse causality cannot be ruled out. Patients who are dissatisfied with their RGP may be more likely to seek care from other GPs, thereby reducing continuity and contributing to the observed associations between continuity of care and patient-reported experiences. However, the RGP scheme in Norway assigns patients to one RGP. While patients can apply to change RGP up to two times per year, provided that the new GP has available capacity, they have limited choice regarding which GP they see for individual consultations. To reduce misclassification due to permanent change of RGP, the eligible study sample included patients who had been listed with the same RGP for ≥12 months prior to the survey, suggesting that GP availability may be more important than patient choice in this setting. We were not able to distinguish between patient choice and GP availability as reasons for consulting other GPs, although patient choice may be less common in the Norwegian system. Future studies may benefit from incorporating information on the reasons for consulting other physicians to better distinguish between these mechanisms. Another limitation is the survey response rate. Although lower response rates can increase the risk of selection bias, evidence suggests that response rates are only weakly related to nonresponse bias in large-scale surveys [38]. The proportion of women was higher among respondents than in the invited sample. This may reflect the inclusion criteria requiring at least three GP consultations, as women typically have higher consultation rates in general practice [39]. Compared with the eligible study sample, survey respondents were slightly older, more often Norwegian-born, and more highly educated. Although all variables differed statistically, absolute differences were small. In addition, UPC scores were approximately comparable between survey respondents and the eligible study sample, indicating negligible effects of survey non-response.

### Conclusions

This study found a consistent positive association between continuity of care and patient-reported experiences in Norwegian general practice. Both registry-based and self-reported continuity were associated with better patient-reported experiences, with stronger associations for self-reported continuity. The associations were stronger among patients with long-term conditions, highlighting the importance of continuity for this group. The asymmetric association between the two continuity measures indicates that they capture related but non-interchangeable aspects of continuity. Combining registry-based and patient-reported experience data provides a more robust basis for monitoring continuity and for informing policy and practice in primary care.

## Supplementary Material

cmag025_Supplementary_Data

## Data Availability

The datasets generated during and/or analysed during the current study are available from the corresponding author on reasonable request.
