# Supplementary material for: Continuity of care and patient-reported experiences in Norwegian general practice: are they linked, and does the measurement of continuity matter?
Source: Fam Pract. 2026 May 13;43(3):cmag025. doi: 10.1093/fampra/cmag025 (PMC13168889; doi:10.1093/fampra/cmag025)
Supplement: cmag025_Supplementary_Data [file cmag025_supplementary_data.pdf]

Table S1. Associations between continuity of care and patient-reported experience scales (0–100; higher = better), stratified by long-term condition status (No vs Yes [a])

	Assessment of GP [b]		Accessibility [b]		Enablement [b]	
	No	Yes	No	Yes	No	Yes
	Coefficient [95% CI]	Coefficient [95% CI]	Coefficient [95% CI]	Coefficient [95% CI]	Coefficient [95% CI]	Coefficient [95% CI]
**Medium UPC (ref. High)** [b]	-2.05 [-2.85, -1.25]	-3.42 [-3.86, -2.98]	-4.94 [-6.20, -3.68]	-6.77 [-7.46, -6.08]	-2.14 [-3.22, -1.05]	-4.39 [-4.93, -3.84]
**Low UPC (ref. High)** [b]	-4.97 [-5.97, -3.96]	-7.51 [-8.10, -6.93]	-7.00 [-8.58, -5.42]	-10.85 [-11.77, -9.93]	-5.38 [-6.77, -4.00]	-8.36 [-9.09, -7.62]
**Other GPs (ref. own GP)**	-9.64 [-10.62, -8.66]	-13.89 [-14.42, -13.36]	-13.66 [-15.22, -12.09]	-17.46 [-18.30, -16.61]	-11.14 [-12.49, -9.78]	-14.77 [-15.44, -14.10]

[a] *Yes = one or more long-term conditions.*

[b] *UPC values are categorised as low: ≤ 0.4; medium: 0.40–0.7; high: > 0.7.*

[c] *Scales 0–100 (higher = better).*

Negative coefficients indicate lower scores compared with the reference group (high UPC / own GP).

Models were adjusted for age, sex, education, household income, region of birth, municipality, and self-reported physical and mental health.

All coefficients were statistically significant (p < 0.001); 95% confidence intervals are shown.
